# Stimulated Brillouin scattering of backward stimulated Raman scattering

**DOI:** 10.1038/s41598-020-59727-4

**Published:** 2020-02-26

**Authors:** Qingsong Feng, Lihua Cao, Zhanjun Liu, Chunyang Zheng, Xiantu He

**Affiliations:** 1 Institute of Applied Physics and Computational Mathematics, Beijing, 100094 China; 2 HEDPS, Center for Applied Physics and Technology, Peking University, Beijing, 100871 China; 3 Collaborative Innovation Center of IFSA (CICIFSA), Shanghai Jiao Tong University, Shanghai, 200240 China

**Keywords:** Laser-produced plasmas, Plasma physics

## Abstract

The rescattering of backward stimulated Raman scattering (BSRS) by stimulated Brillouin scattering (SBS) is found in the high electron density region by relativistic Vlasov-Maxwell simulation and particle-in-cell (PIC) simulation, where the BSRS is in the regime of absolute instability and dominates in all the scatterings. Both one dimension (1D) Vlasov simulation and two dimension (2D) PIC simulation have been given to verify that there exists SBS of BSRS in the regime of absolute instability for BSRS. The SBS of BSRS will be even stronger than forward stimulated Raman scattering (FSRS) and SBS in regime of absolute instability for BSRS. Thus, besides Langmuir decay instability and laser energy absorption, the SBS of BSRS is also an important saturation mechanism of BSRS in high electron density region.

## Introduction

In inertial confinement fusion (ICF)^1–3^, the main parametric decay instabilities include stimulated Raman scattering (SRS) and stimulated Brillouin scattering (SBS). SRS^4^ is a three-wave interaction process where an incident light couples with a forward-propagating Langmuir wave (LW) to produce a backward scattering light (BSRS) or forward scattering light (FSRS). And SBS^5^ is a three-wave interaction process where an incident light couples with an ion-acoustic wave (IAW) to produce a backscattering light. BSRS and SBS will lead to a great energy loss of incident laser. And BSRS or FSRS will produce a number of hot electrons to preheat fusion fuel, which will have a detrimental effect on the symmetrical compression of fusion capsule. Thus, the suppression of SRS and SBS is an important component of the laser-driven ICF research.

The possible saturation mechanisms for BSRS are trapped-particle instability^6^, Langmuir decay instability (LDI)^7–14^, Langmuir collapse^15–18^, or nonlinear frequency shift due to particle trapping^19–22^ and so on. Many mechanisms for the saturation of SBS have been proposed, such as increasing linear Landau damping by kinetic ion heating^23,24^, the creation of cavities in plasmas^25,26^, frequency detuning due to particle trapping^27–29^, coupling with higher harmonics^30–32^ and so on. The rescattering in laser plasma interaction is also an important saturation mechanism of SRS or SBS. Especially, SBS cascade scattering as a saturation mechanism of SBS in high-intensity laser-plasma interaction had been researched by Feng *et al*.^33^ And Winjum *et al*.^34^ researched the role of BSRS of BSRS, BSRS of FSRS in superthermal electron generation if the electron density was lower than $\sim 0.1{n}_{c}$ (${n}_{c}$ is the critical density of incident laser). SBS of FSRS had been found by Langdon *et al*.^35^ and been observed in experiment by Hinkel *et al*.^36^ in the condition of high electron temperature, where the FSRS would be much stronger than BSRS. However, in moderate electron density such as ${n}_{e}=0.1-0.2{n}_{c}$, which is closer to the ICF experiment condition, the BSRS will dominate in all scatterings. Thus, the rescattering of BSRS by SBS may occur if the BSRS is strong enough.

In this paper, we report the first demonstration that SBS of BSRS will exist especially in the high electron density region, such as ${n}_{e} \sim 0.2{n}_{c}$, where the BSRS is in the regime of absolute instability and will dominate in all the scatterings. Through 1D relativistic Vlasov-Maxwell simulation and 2D PIC simulation, the subtle spectrum of SBS of BSRS has been distinguished from FSRS. And in regime of absolute instability for BSRS, the SBS of BSRS will be even stronger than FSRS and SBS excited by the incident laser. These results illustrate that besides LDI and laser energy absorption, the SBS of BSRS is also an important saturation mechanism of BSRS in high electron density region.

## Results

### Theoretical analyses

**Figure 1:**
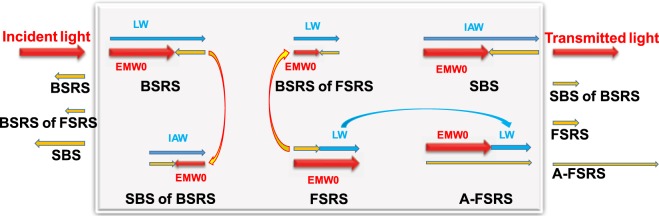
The schematic diagram of the main three-wave relations in laser-plasma interaction.

 Figure 1 gives a schematic of main three-wave instabilities, such as BSRS, SBS, FSRS and rescatterings. Especially, if the BSRS is strong enough, the BSRS scattering light will excite SBS as a pump light. For example, the BSRS is in the regime of absolute instability in condition of ${n}_{e}=0.2{n}_{c}$, thus the BSRS is a strong pump light to excite SBS. If ${n}_{e} < 0.108{n}_{c}$ in condition of ${T}_{e}=2.5keV$, the electron density corresponding to critical density ${n}_{c}^{F}$ of FSRS scattering light is slightly lower than $0.25{n}_{c}^{F}$, thus BSRS of FSRS will exist. However, if ${n}_{e} > 0.108{n}_{c}$, the electron density corresponding to critical density ${n}_{c}^{F}$ of FSRS scattering light is larger than $0.25{n}_{c}^{F}$, thus, BSRS of FSRS can not exist. In the same way, BSRS of BSRS will exist in condition of ${n}_{e} < 0.1{n}_{c}$. The LW generated by FSRS will couple with the incident pump light to generate a scattering light with higher frequency than the pump light, which is called anti-Stokes FSRS (A-FSRS) process. In our simulation, ${n}_{e}=0.2{n}_{c}$ and ${n}_{e}=0.1{n}_{c}$ are taken as typical parameters to simulate rescatterings of BSRS. Thus, there are scattering lights of BSRS, BSRS of FSRS and SBS from the left boundary, and SBS of BSRS, FSRS, A-FSRS from the right boundary.

Under the envelope approximation, coupling of SRS and SBS can be described by linear five-waves interaction equations in homogenous plasmas^5,37,38^:

1 $$(\frac{\partial }{\partial t}+{v}_{g0}\frac{\partial }{\partial x}+{\nu }_{0}){A}_{0}(x,t)=-i\frac{\pi {e}^{2}}{{\omega }_{0}{m}_{e}}(\delta {n}_{L}{A}_{R}+\delta {n}_{A}{A}_{B}),$$

2 $$(\frac{\partial }{\partial t}-{v}_{gR}\frac{\partial }{\partial x}+{\nu }_{R}){A}_{R}(x,t)=-i\frac{\pi {e}^{2}}{{\omega }_{R}{m}_{e}}\delta {n}_{L}^{* }{A}_{0},$$

3 $$(\frac{\partial }{\partial t}-{v}_{gB}\frac{\partial }{\partial x}+{\nu }_{B}){A}_{B}(x,t)=-i\frac{\pi {e}^{2}}{{\omega }_{B}{m}_{e}}\delta {n}_{A}^{* }{A}_{0},$$

4 $$(\frac{\partial }{\partial t}+{v}_{gL}\frac{\partial }{\partial x}+{\nu }_{L})\delta {n}_{L}(x,t)=-i\frac{{n}_{e}{e}^{2}{k}_{L}^{2}}{4{\omega }_{L}{m}_{e}^{2}{c}^{2}}{A}_{0}{A}_{R}^{* },$$

5 $$(\frac{\partial }{\partial t}+{v}_{gA}\frac{\partial }{\partial x}+{\nu }_{A})\delta {n}_{A}(x,t)=-i\frac{{\bar{Z}}_{i}{n}_{e}{e}^{2}{k}_{A}^{2}}{4{\omega }_{A}{m}_{e}{\bar{m}}_{i}{c}^{2}}{A}_{0}{A}_{B}^{* },$$

where ${A}_{0}(x,t)$, ${A}_{R}(x,t)$ and $\delta {n}_{L}(x,t)$ are the complex amplitudes of the vector potentials of the pump light, SRS scattering light, and LW. ${A}_{B}(x,t)$ and $\delta {n}_{A}(x,t)$ are the complex amplitudes of the vector potentials of SBS backscattering light and IAW. And ${\nu }_{i}$, ${v}_{gi}$, ${\omega }_{i}$ are damping rates, group velocities and frequencies of the pump light ($i=0$), SRS scattering light ($i=R$), SBS scattering light ($i=B$), LW ($i=L$), and IAW ($i=A$). From the five-waves linear equations, we can derive the thresholds, growth rates and gains of SRS and SBS.

In homogeneous plasmas, the threshold of SRS is

6 $${\gamma }_{thR}=\sqrt{{\nu }_{L}{\nu }_{R}},$$

and the threshold of SBS is

7 $${\gamma }_{thB}=\sqrt{{\nu }_{A}{\nu }_{B}},$$

where $${\nu }_{L},{\nu }_{R},{\nu }_{A},{\nu }_{B}$$ are the damping of Langmuir wave, SRS scattering light, IAW, SBS scattering light. Only Landau dampings of LW $${\nu }_{L}=\sqrt{\pi /8}{\omega }_{L}{(k{\lambda }_{De})}^{-3}\,{\rm{\exp }}\,[-1/{(\sqrt{2}{k}_{L}{\lambda }_{De})}^{2}-3/2]$$ and IAW $${\nu }_{A}=\sqrt{\pi /8}{\omega }_{A}[{({Z}_{i}{m}_{e}/{m}_{i})}^{1/2}+$$$${({Z}_{i}{T}_{e}/{T}_{i})}^{3/2}\,{\rm{\exp }}\,(-{Z}_{i}{T}_{e}/(2{T}_{i})-3/2)]$$ are considered, since the Landau damping is much larger than collision damping in our simulation. And $${\nu }_{R}={\omega }_{pe}^{2}{\nu }_{ei}/(2{\omega }_{R}^{2})$$, $${\nu }_{B}={\omega }_{pe}^{2}{\nu }_{ei}/(2{\omega }_{B}^{2})$$, where $${\nu }_{ei}$$ is the electron-ion collision frequency. If the maximum temporal growth rate of SRS^5,38^

8 $${\gamma }_{0R}=\frac{1}{4}\sqrt{\frac{{\omega }_{pe}^{2}}{{\omega }_{R}{\omega }_{L}}}{k}_{L}{a}_{0}$$

and the maximum temporal growth rate of SBS^39,40^

9 $${\gamma }_{0B}=\frac{1}{4}\sqrt{\frac{{n}_{e}}{{n}_{c}}}\frac{{a}_{0}}{{v}_{te}}\sqrt{{\omega }_{0}{\omega }_{A}}$$

are larger than the thresholds of SRS and SBS, the SRS and SBS will be excited. Where ${\omega }_{i}$, ${k}_{i}$ are the frequency and wave number of LW ($i=L$), IAW ($i=A$), SRS scattering light ($i=R$) and SBS scattering light ($i=B$). ${a}_{0}=eE/{m}_{e}{\omega }_{0}$ is the quiver velocity of electron. In the same way, if the BSRS scattering light as a pump light makes the maximum temporal growth rate larger than the threshold of SBS, the SBS of BSRS will be excited. The gain of SRS is:

10 $${G}_{R}=2\frac{{\gamma }_{0R}^{2}}{{\nu }_{L}{v}_{gR}}L,$$

and the gain of SBS is:

11 $${G}_{B}=2\frac{{\gamma }_{0B}^{2}}{{\nu }_{A}{v}_{gB}}L,$$

where $L$ is the plasma density scale length, ${v}_{gi}$ is the group velocity of SRS ($i=R$) and SBS ($i=B$) scattering light.

**Figure 2:**
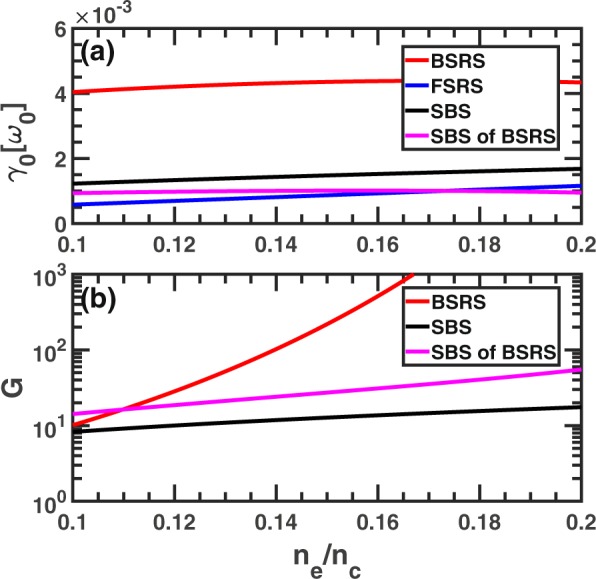
The maximum temporal growth rate and gain of BSRS, FSRS, SBS and SBS of BSRS. The gain of FSRS is not shown here due to very large value, if only the Landau damping of LW is considered. Where $${a}_{0}$$ is assumed to be the incident light amplitude with no pump depletion. The condition is $${n}_{e}=0.2{n}_{c},{I}_{0}=3\times 1{0}^{15}W/c{m}^{2},{L}_{x}$$$$=\,5000c/{\omega }_{0}$$ as the same with case 3.

The maximum temporal growth rate and gain of BSRS, FSRS, SBS and SBS of BSRS have been shown in Fig. 2. If the pump light amplitude is assumed to be incident light amplitude, the growth rate of SBS will be larger than that of the SBS of BSRS. And the growth rate of BSRS is much larger than that of FSRS, SBS and SBS of BSRS, thus the BSRS will dominate in all the scatterings in the condition of ${n}_{e}$ from $0.1{n}_{c}$ to $0.2{n}_{c}$. However, if BSRS scattering light is strong enough and the pump depletion of incident light is strong enough, the BSRS scattering light as a pump light will be stronger than the pump light of SBS, thus BSRS will excited SBS with amplitude larger than SBS excited by the incident light.

### Vlasov simulations

**Figure 3:**
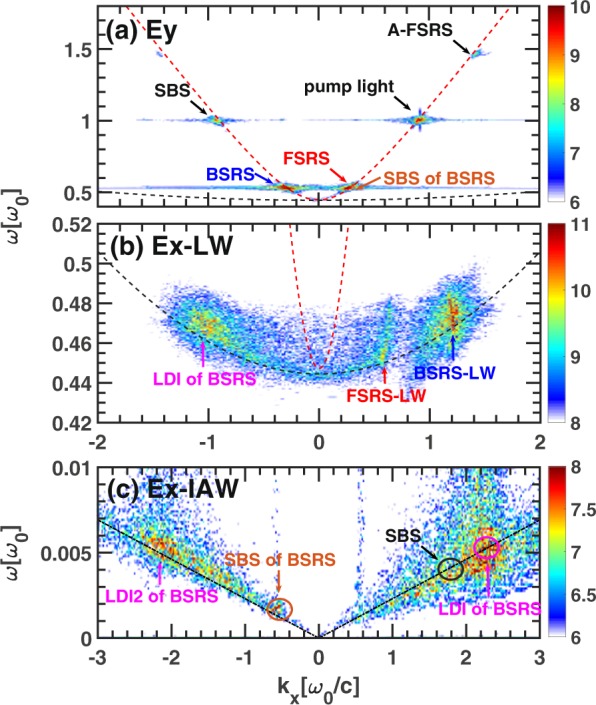
The dispersion relation of electromagnetic waves and electrostatic waves in the condition of case 1. The dispersion relation of (**a**) transverse electric field ${E}_{y}$ and (**b**), (**c**) longitudinal electric field ${E}_{x}$. The parameters are case 1: ${n}_{e}=0.2{n}_{c},{I}_{0}=1\times 1{0}^{16}W/c{m}^{2},{L}_{x}=500c/{\omega }_{0}$.

To show the SBS of BSRS process, the dispersion relations of electromagnetic waves and electrostatic waves are shown in Fig. 3. We can see that the frequencies of FSRS and SBS of BSRS are very close to each other. We can distinguish these two scatterings by the products of FSRS and SBS of BSRS. The LW produced by FSRS is very clear as shown in Fig. 3(b), and the IAW produced by SBS of FSRS can be seen in Fig. 3(c). Since the wave number of LW generated by BSRS ${k}_{L}^{B}{\lambda }_{De}=0.18$ is small in condition of ${n}_{e}=0.2{n}_{c},{T}_{e}=2.5keV$, the LDI cascade will occur^9,14,15^. The corresponding LWs and IAWs generated by LDI cascade are shown in Fig. 3(b,c). The LDI will dissipate the energy of LW generated by BSRS to decay LW and IAW, thus saturating BSRS.

**Figure 4:**
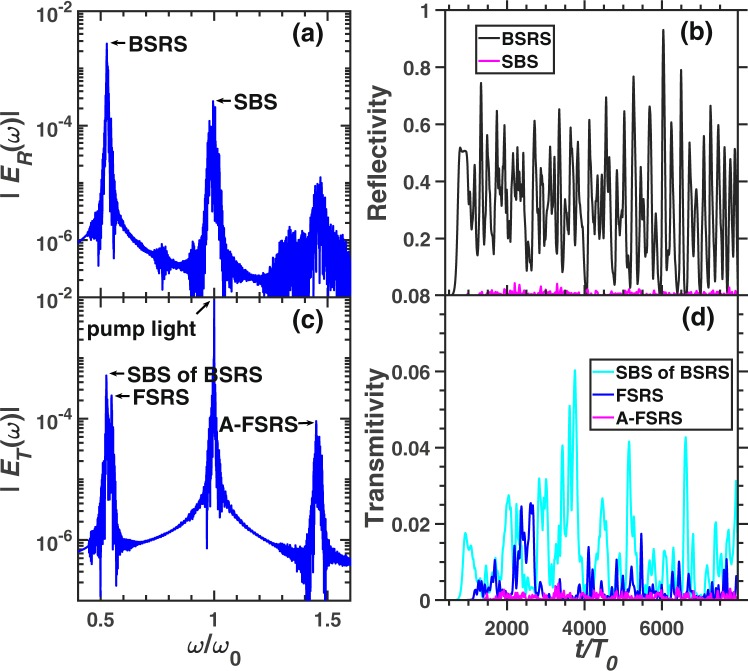
The spectra and scattering rate of each scattering in the condition of case 2. The frequency spectra of (**a**) reflective light electric field ${E}_{R}$ at the left boundary (incident boundary) and (**c**) transmitting light electric field ${E}_{T}$ at the right boundary (transmitting boundary). (**b**) The reflectivity of BSRS $([0.45,0.6]{\omega }_{0})$ and SBS $([0.9,1.1]{\omega }_{0})$. (**d**) The scattering rate of FSRS $([0.535,0.6]{\omega }_{0})$, SBS of BSRS $([0.45,0.535]{\omega }_{0})$, and A-FSRS $([1.4,1.5]{\omega }_{0})$. The condition is case 2: ${n}_{e}=0.2{n}_{c},{I}_{0}=3\times 1{0}^{15}W/c{m}^{2},{L}_{x}=500c/{\omega }_{0}$.

 Figure 4 demonstrates the frequency spectra and scattering rates of BSRS, SBS, FSRS and rescatterings in case 2. The frequency of each scattering light is consistent to the theoretical value, which is listed in Table 1. The BSRS will dominate and is much stronger than FSRS in the condition of ${n}_{e}=0.2{n}_{c},{T}_{e}=2.5keV$. Since the spatial scale ${L}_{x}=500c/{\omega }_{0}=28\ \mu m$ is small, the FSRS can not be excited to a large level. The strong BSRS scattering light will excite SBS with amplitude larger than FSRS as shown in Fig. 4(d). Among the simulation time scale, the average scattering rates are as follows: BSRS: $28.64 \%$, SBS: $0.54 \%$, SBS of BSRS: $1.05 \%$, FSRS: $0.28 \%$, A-FSRS: $0.06 \%$, the transmitivity is $41.04 \%$ and the absorption rate is $28.46 \%$. The laser energy absorption mainly comes from that the BSRS-induced LW accelerates electrons and BSRS-induced LW decays into a LW and IAW by LDI. Thus the pump light or BSRS scattering light will transfer energy to hot electrons through electron trapping and decay products through LDI. The intensity of SBS of BSRS is stronger than SBS and FSRS. Thus, besides the LDI cascade and laser energy absorption, the SBS of BSRS can also saturate BSRS.

**Table 1:** The simulation parameters and frequencies of SRS, SBS, A-FSRS and rescatterings.

Conditions					Scattering lights from left boundary				Transmitted lights from right boundary		
case	*ne*[*nc*]	*I0*[$1{0}^{15}W/c{m}^{2}$]	$Lx$[$c/{\omega }_{0}$]	ions	$\omega [{\omega }_{0}]$	BSRS of FSRS	BSRS	SBS	SBS of BSRS	FSRS	A-FSRS
1	0.2	10	500	mobile	Simulation	$\backslash$	0.525	0.9953	0.529(h)${}^{a}$	0.5434(l)${}^{b}$	1.451
					Theory	$\backslash$	0.5305	0.9959	0.5237${}^{c}$	0.55	1.453
2	0.2	3	500	mobile	Simulation	$\backslash$	0.5269	0.9953	0.5246(h)	0.5479(l)	1.45
					Theory	$\backslash$	0.5305	0.9959	0.5256	0.55	1.453
3	0.2	3	5000	mobile	Simulation	$\backslash$	0.525	0.9968	0.5238(l)	0.547(h)	1.45
					Theory	$\backslash$	0.5305	0.9959	0.5237	0.55	1.453
4	0.1	3	5000	mobile	Simulation	0.326	0.659	0.9958	0.6379(l)	0.6813(h)	1.318
					Theory	0.3518	0.635	0.9956	0.6559	0.681	1.319
5	0.1–0.2	3	5000	mobile	Simulation	$\backslash$	0.537–0.627	0.9956	$\backslash$	0.560–0.614	1.368–1.439
					Theory	$\backslash$	0.531–0.635	0.9956	$\backslash$	0.55–0.681	1.319–1.453
6	0.2	3	500	fixed	Simulation	$\backslash$	0.5292	$\backslash$	$\backslash$	0.5483	1.449
					Theory	$\backslash$	0.5305	$\backslash$	$\backslash$	0.55	1.453

The conditions T_e_ = 2.5 keV, T_i_ = 1/3T_e_ are the same in all simulation cases.

^a^ h refers to “higher” amplitude than FSRS.

^b^ l refers to “lower” amplitude than SBS of BSRS.

^c^ The BSRS from simulation is taken as a pump light to calculate the theoretical frequency of SBS of BSRS.

**Figure 5:**
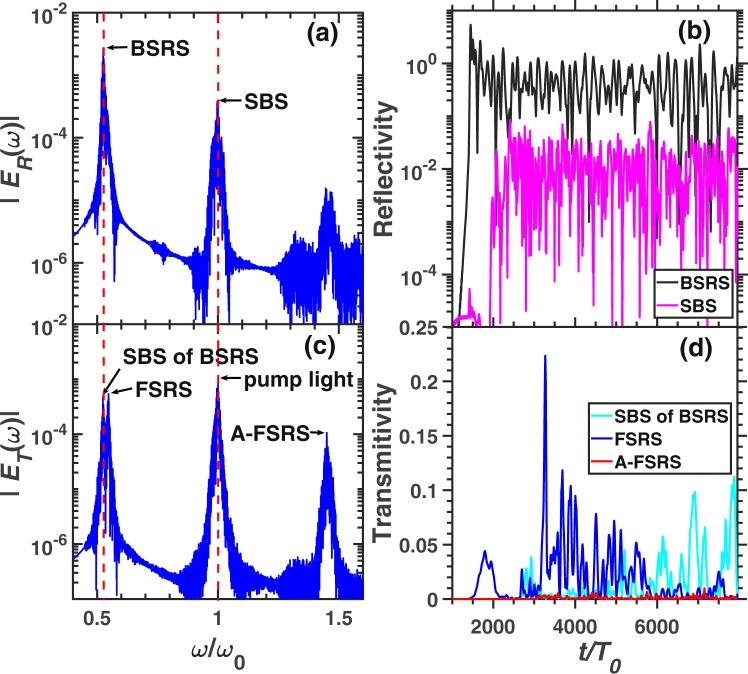
The spectra and scattering rate of each scattering in the condition of case 3. The frequency spectra of (**a**) reflective light electric field $${E}_{R}$$ and (**c**) transmitting light electric field $${E}_{T}$$. (**b**) The reflectivity of BSRS $$([0.45,0.6]{\omega }_{0})$$ and SBS $$([0.9,1.1]{\omega }_{0})$$. (**d**) The scattering rate of FSRS $$([0.535,0.6]{\omega }_{0})$$, SBS of BSRS $$([0.45,0.535]{\omega }_{0})$$, and A-FSRS $$([1.4,1.5]{\omega }_{0})$$. The condition is case 3: $${n}_{e}=0.2{n}_{c},{I}_{0}=3\times 1{0}^{15}W/c{m}^{2},{L}_{x}$$$$=5000c/{\omega }_{0}$$.

 Figure 5 shows a larger scale simulation. BSRS and SBS will be scattered from the left boundary, while the SBS of BSRS, FSRS and A-FSRS will be scattered from the right boundary. The frequency of SBS of BSRS from simulation is ${\omega }_{s}=0.5238{\omega }_{0}$, which is very close to the theoretical value ${\omega }_{t}=0.5237{\omega }_{0}$. Thus, the spectrum is indeed SBS of BSRS. Another spectrum with a frequency slightly higher than that of the SBS of BSRS is ${\omega }_{s}=0.547{\omega }_{0}$, which is close to the theoretical value of FSRS ${\omega }_{t}=0.55{\omega }_{0}$. From Fig. 5(c), the intensities of SBS of BSRS and FSRS are comparable. As shown in Fig. 5(d), the FSRS will dominate before $t\simeq 6000{T}_{0}$, and SBS of BSRS will be stronger than FSRS after $t\simeq 6000{T}_{0}$ and dominate among all the scattering lights from the right boundary. The average scattering rate among the total simulation time of each scattering is as follows: BSRS: $42.73 \%$, SBS: $1.03 \%$, SBS of BSRS: $1.14 \%$, FSRS: $1.57 \%$, A-FSRS: $0.07 \%$, the transmitivity: $6.16 \%$ and absorption rate: $47.29 \%$. The large scale plasma (${L}_{x}=280\ \mu m$) with high electron density ${n}_{e}=0.2{n}_{c}$ will absorb a large amount of the pump laser energy through electrons accelerated by LW and LDI cascade. The A-FSRS is very weak which is negligible. The BSRS dominates in all scatterings. However, the SBS, SBS of BSRS and FSRS are comparable. Since the pump depletion due to strong BSRS and laser energy absorption, the pump light will be weaker than the BSRS scattering light. Thefore, the SBS of BSRS is slightly stronger than SBS, which will play an important role in saturation of BSRS.

### PIC simulations

**Figure 6:**
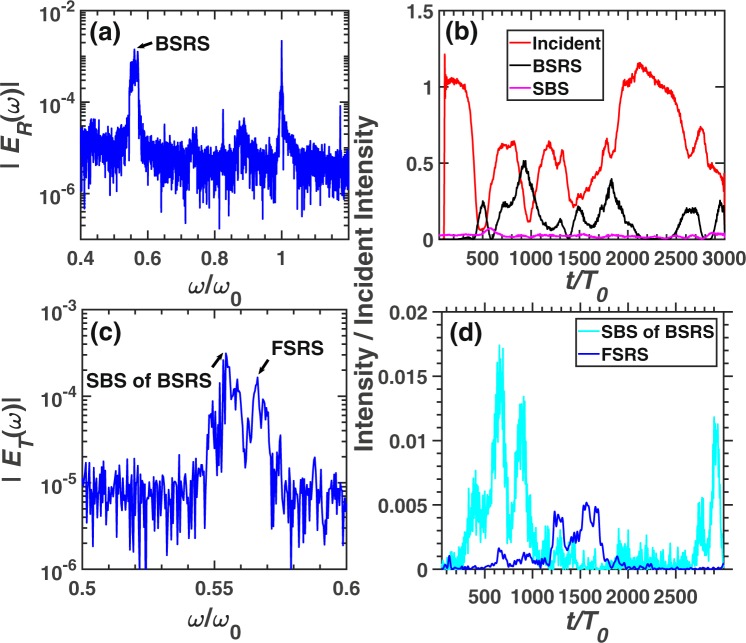
The spectra and scattering rate of each scattering in condition of ${L}_{x}=100{\lambda }_{0}$ from 2D PIC simulation. The frequency spectra of (**a**) reflective light electric field ${E}_{R}$ at the left boundary (incident boundary) and (**c**) transmitting light electric field ${E}_{T}$ at the right boundary (transmitting boundary). (**b**) The reflectivity of BSRS $([0.45,0.6]{\omega }_{0})$ and SBS $([0.9,1.1]{\omega }_{0})$. (**d**) The scattering rate of FSRS $([0.559,0.6]{\omega }_{0})$, and SBS of BSRS $([0.45,0.559]{\omega }_{0})$. The condition is: ${n}_{e}=0.2{n}_{c},{I}_{0}=3\times 1{0}^{15}W/c{m}^{2},{L}_{x}=100{\lambda }_{0}$, ${L}_{y}=40{\lambda }_{0}$.

To verify that there exists SBS of BSRS in 2D system, a short scale 2D PIC simulation with ${L}_{x}=100{\lambda }_{0}$ is conducted. The central axis of laser propagation direction $y=0\in [-{l}_{y}/2,{l}_{y}/2]$ is chosen to give the scattering rate and transmitivity. As shown in Fig. 6, BSRS will be excited to a large amplitude with maximum amplitude of $\sim 50 \%$. At the same time, SBS of BSRS will be excited to a maximum amplitude of $\sim 1.5 \%$. The frequency of BSRS scattering light is $0.5603{\omega }_{0}$ and SBS of BSRS scattering light is $0.5543{\omega }_{0}$, while the FSRS scattering light is $0.5663{\omega }_{0}$. The theoretical frequency of SBS of BSRS is $0.559{\omega }_{0}$, which is close to the simulation value $0.554{\omega }_{0}$. The frequencies of BSRS and FSRS scattering lights are slightly higher than the theoretical values, because electron density will decrease slightly in the central axis of laser propagation in 2D simulation. The average scattering rate among the total simulation time of each scattering is as follows: BSRS: $11.94 \%$, SBS: $0.12 \%$, SBS of BSRS: $0.25 \%$, FSRS: $0.084 \%$, transmitivity: $61.02 \%$, and absorption rate: $26.7 \%$. We can see that the average scattering rate of SBS of BSRS is much larger than that of FSRS or SBS. This illustrates that SBS of BSRS also plays an important role in the saturation of BSRS in 2D system.

**Figure 7:**
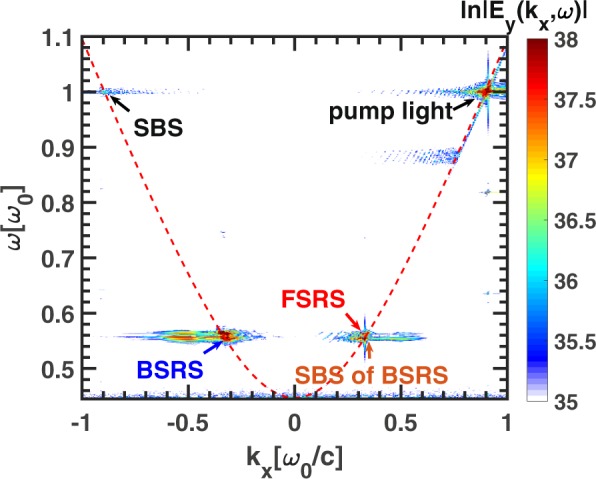
The dispersion relation of electromagnetic waves ${E}_{y}$ in the condition of ${L}_{x}=200{\lambda }_{0}$ from 2D PIC simulation. The parameters are: ${n}_{e}=0.2{n}_{c},{I}_{0}=3\times 1{0}^{15}W/c{m}^{2},{L}_{x}=200{\lambda }_{0}$, ${L}_{y}=40{\lambda }_{0}$.

**Figure 8:**
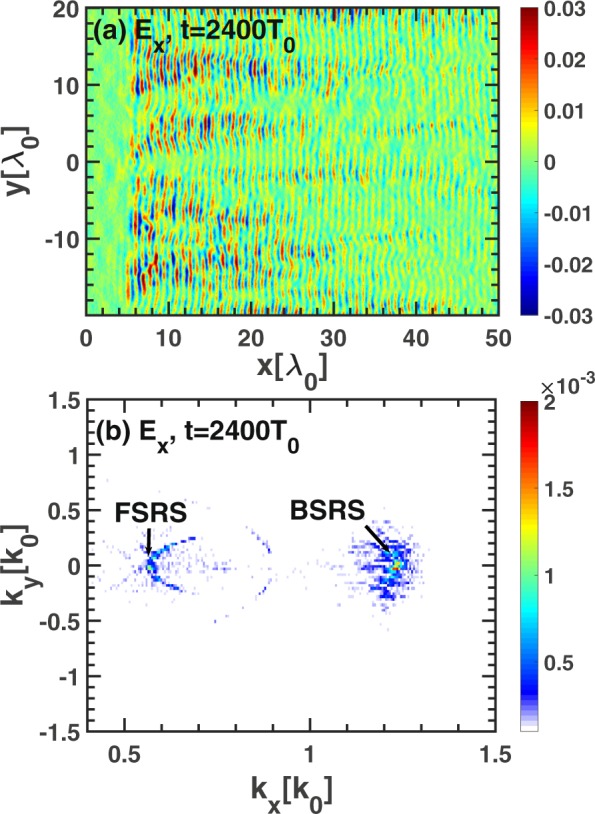
The spatial distribution (**a**) and spatial spectrum (**b**) of ${E}_{x}$ under the condition of ${L}_{x}=200{\lambda }_{0}$ from 2D PIC simulation. The condition is: ${n}_{e}=0.2{n}_{c},{I}_{0}=3\times 1{0}^{15}W/c{m}^{2},{L}_{x}=200{\lambda }_{0}$, ${L}_{y}=40{\lambda }_{0}$.

A larger scale with ${L}_{x}=200{\lambda }_{0}$ simulation has been conducted. As shown in Fig. 7, the spectrum of ${E}_{y}(x,y=0,t)$ is ${E}_{y}({k}_{x},\omega )$. The SBS and BSRS will occur and the BSRS will dominate in all the scatterings, and at the same time the SBS of BSRS and FSRS will occur, which are labelled in Fig. 7. The spatial distribution and spatial spectrum of ${E}_{x}$ at a fixed time $t=2400{T}_{0}$ is shown in Fig. 8. In 2D plasma system, the 2D kinetic effects such as wave bowing^41,42^, transverse localization^43^, and filamentation^44^ may affect the BSRS LW. The wave bowing results from the nonlinear dispersion associated with the nonlinear frequency shift^45^ of LW during SRS. Since the negative nonlinear frequency shift of LW increases with increasing wave amplitude, the wave phase velocity decreases with increasing wave amplitude. If the laser envelope is a 2D Gaussian as shown by Yin *et al*.^42^, smaller-amplitude waves offset from the center of the laser speckle travel faster in x than do larger-amplitude waves at the center of the speckle. As a result, the wavefronts bend^42^. However, in our 2D simulation, the laser envelope is a 2D plane with the same intensity in y direction. Thus, the wave bowing and even transverse breakup of Langmuir wave are not obvious. Since the Langmuir wave excited by SRS does not uniformly distribute in the y direction due to 2D kinetic effects such as transverse localization^43^ and filamentation^44^, the local wave bowing will occur as shown in Fig. 8(a). The stronger LW will bend in the negative x-direction, which is consistent to the negative nonlinear frequency shift of LW^45^. Although there exist 2D kinetic effects on BSRS LW, which is not obvious due to the plane pump laser taken in our simulation. And SBS of BSRS can also play an important role in saturation of BSRS even though there exist 2D kinetic effects on BSRS LW, which will be shown latter. As shown in Fig. 8(b), the spatial spectrum of ${E}_{x}$ can clearly demonstrate FSRS and BSRS LW wave numbers. The wave number of BSRS LW obtained from Fig. 8(b) is ${k}_{L}^{B}=1.23{\omega }_{0}/c$, which is close to theoretical value $1.18{\omega }_{0}/c$. And the wave number of FSRS LW from Fig. 8(b) is ${k}_{L}^{F}=0.572{\omega }_{0}/c$, which is close to the theoretical value $0.58{\omega }_{0}/c$. This clearly illustrates that the BSRS and FSRS will coexist.

**Figure 9:**
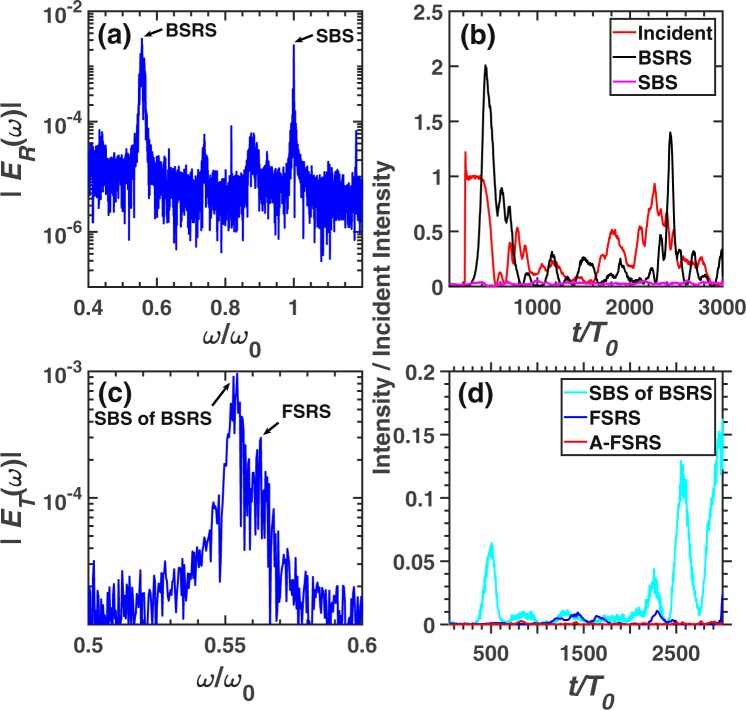
The spectra and scattering rate of each scattering in the condition of ${L}_{x}=200{\lambda }_{0}$ from 2D PIC simulation. The frequency spectra of (**a**) reflective light electric field ${E}_{R}$ at the left boundary (incident boundary) and (**c**) transmitting light electric field ${E}_{T}$ at the right boundary (transmitting boundary). (**b**) The reflectivity of BSRS $([0.45,0.6]{\omega }_{0})$ and SBS $([0.9,1.1]{\omega }_{0})$. (**d**) The scattering rate of FSRS $([0.559,0.6]{\omega }_{0})$, SBS of BSRS $([0.45,0.559]{\omega }_{0})$, and A-FSRS $([1.4,1.5]{\omega }_{0})$. The condition is: ${n}_{e}=0.2{n}_{c},{I}_{0}=3\times 1{0}^{15}W/c{m}^{2},{L}_{x}=200{\lambda }_{0}$, ${L}_{y}=40{\lambda }_{0}$.

As shown in Fig. 9, there exist BSRS, SBS, SBS of BSRS and FSRS. The spectrum with frequency close to ${\omega }_{0}$ contains the SBS scattering light and reflected light of pump light. From Fig. 9(a,c), the frequency of BSRS scattering light is $0.5563{\omega }_{0}$ and SBS of BSRS scattering light is $0.5543{\omega }_{0}$, while the FSRS scattering light is $0.563{\omega }_{0}$. The theoretical frequency of SBS of BSRS is $0.5548{\omega }_{0}$, which is very close to the simulation value $0.5543{\omega }_{0}$. This illustrates that there also exists SBS of BSRS in 2D plasma system. The frequencies of BSRS and FSRS scattering lights are slightly higher than the theoretical values, because electron density will decrease slightly in the central axis of laser propagation in 2D simulation. The average scattering rate among the total simulation time of each scattering is as follows: BSRS: $25.12 \%$, SBS: $0.16 \%$, SBS of BSRS: $2.05 \%$, FSRS: $0.20 \%$, transmitivity: $30.0 \%$, and absorption rate: $42.67 \%$. Since the LW induced by BSRS will trap electrons and transfer energy from LW to electrons, and LDI cascade will transfer energy from BSRS LW to decay LW and IAW, thus the absorption rate is higher with larger scale plasma. We can see that the average scattering rate from SBS of BSRS is much larger than that from FSRS or SBS. Therefore, absorption of laser and SBS of BSRS are both important saturation mechanisms of BSRS.

**Figure 10:**
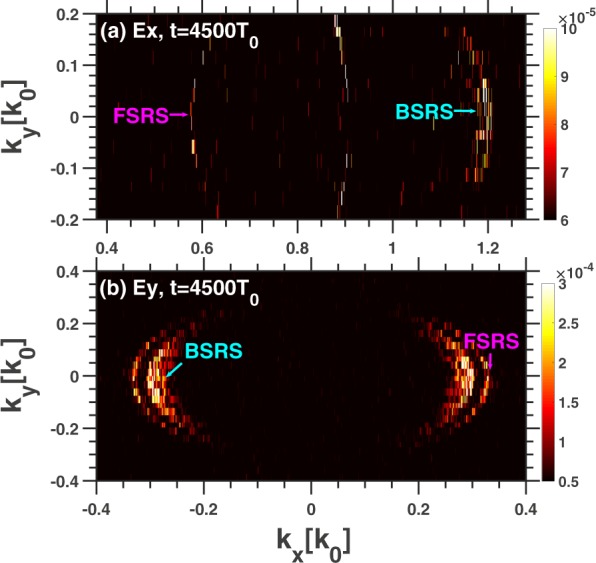
The spatial spectra of (**a**) $${E}_{x}$$ and (**b**) $${E}_{y}$$. The condition is: $${n}_{e}=0.2{n}_{c},{I}_{0}=3\times 1{0}^{15}W/c{m}^{2},{L}_{x}$$$$=1000{\lambda }_{0}$$, $${L}_{y}=40{\lambda }_{0}$$.

A large scale 2D PIC simulation has been conducted with ${L}_{x}=1000{\lambda }_{0}$ and ${L}_{y}=40{\lambda }_{0}$. As shown in Fig. 10(a), the wave number of BSRS LW is ${k}_{L}^{B}=1.188{\omega }_{0}/c$, which is close to the theoretical value $1.18{\omega }_{0}/c$, and the wave number of FSRS LW is ${k}_{L}^{F}=0.5774{\omega }_{0}/c$, which is close to the theoretical value $0.58{\omega }_{0}/c$. From Fig. 10(b), the wave number of BSRS scattering light is ${k}_{s}^{B}=-0.298{\omega }_{0}/c$, which is close to the theoretical value $-0.29{\omega }_{0}/c$, and the wave number of FSRS scattering light is ${k}_{s}^{F}=0.328{\omega }_{0}/c$, which is close to the theoretical value $0.32{\omega }_{0}/c$. The spectra are symmetric to the origin, which is from the Fast Fourier Transform (FFT) algorithm. Since the scattering light from SBS of BSRS is nearly symmetric to the BSRS scattering light, SBS of BSRS scattering light can not be distinguished from the symmetric spectrum of BSRS scattering light from FFFT as shown in Fig. 10(b). The strength of BSRS is obviously stronger than that of FSRS, therefore SBS of BSRS can also be easier to occur in large scale plasma. Since the simulation scale is as long as ${L}_{x}=1000{\lambda }_{0}$ and total simulation time is as long as $t=15000{T}_{0}$, the $\omega$ spectrum can not be given due to the limitation of memory capacity. The $\omega$ spectra will be given by large scale 1D PIC simulation, which will be shown latter.

**Figure 11:**
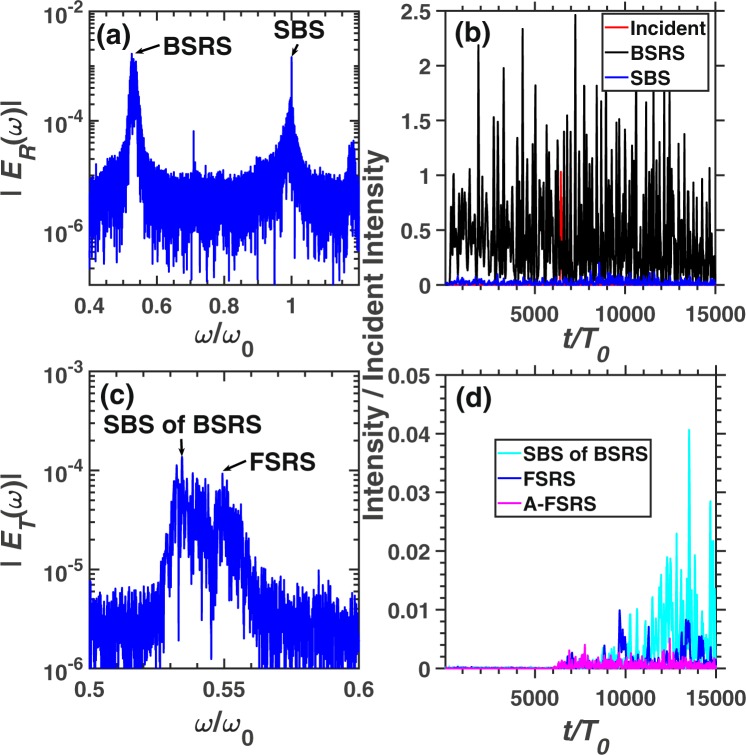
The spectra and scattering rate of each scattering in condition of $${L}_{x}=2020\mu m$$ from 1D PIC simulation. The frequency spectra of (**a**) reflective light electric field $${E}_{R}$$ at the left boundary (incident boundary) and (**c**) transmitting light electric field $${E}_{T}$$ at the right boundary (transmitting boundary). (**b**) The reflectivity of BSRS $$([0.45,0.6]{\omega }_{0})$$ and SBS $$([0.9,1.1]{\omega }_{0})$$. (**d**) The scattering rate of FSRS $$([0.546,0.6]{\omega }_{0})$$, SBS of BSRS $$([0.45,0.546]{\omega }_{0})$$, and A-FSRS $$([1.4,1.5]{\omega }_{0})$$. The condition is: $${n}_{e}=0.2{n}_{c},{I}_{0}=3\times 1{0}^{15}W/c{m}^{2},{L}_{x}$$$$=2020\mu m$$.

As shown in Fig. 11, a large scale 1D PIC simulation has been conducted. The simulation is ${L}_{x}=2020\mu m$ with plasma length ${L}_{p}=2000\mu m$ and $2\times 10\mu m$ vacuum layers in the two sides of plasma boundaries. The total simulation time is as long as ${t}_{end}=1.5\times 1{0}^{4}{T}_{0}=17.55ps$. We can see that there exist BSRS, SBS, SBS of BSRS, and FSRS spectra. From Fig. 11(a,c), the frequency of BSRS scattering light is $0.5379{\omega }_{0}$ (theoretical value: $0.53{\omega }_{0}$) and SBS of BSRS scattering light is $0.5343{\omega }_{0}$, while the FSRS scattering light is $0.5493{\omega }_{0}$ (theoretical value: $0.55{\omega }_{0}$). The theoretical frequency of SBS of BSRS is $0.537{\omega }_{0}$, which is close to the simulation value $0.534{\omega }_{0}$. This illustrates that there also exists SBS of BSRS in large scale ($2\ mm$) plasma system. The frequencies of BSRS and FSRS scattering lights are consistent to theoretical values. The average scattering rate among the total simulation time of each scattering is as follows: BSRS: $43.75 \%$, SBS: $0.81 \%$, SBS of BSRS: $0.22 \%$, FSRS: $0.073 \%$, transmitivity: $0.72 \%$, and absorption rate: $54.5 \%$. Since the LW induced by BSRS will trap and accelerate electrons and transfer huge energy from LW to electrons, and LDI cascade will transfer energy from BSRS LW to decay LW and IAW, thus the absorption rate is very high in large scale ($2mm$) plasmas. We can see that the average scattering rate from SBS of BSRS is much larger than that from FSRS. Therefore, absorption of laser and SBS of BSRS are both important saturation mechanisms of BSRS.

## Discussions

**Figure 12:**
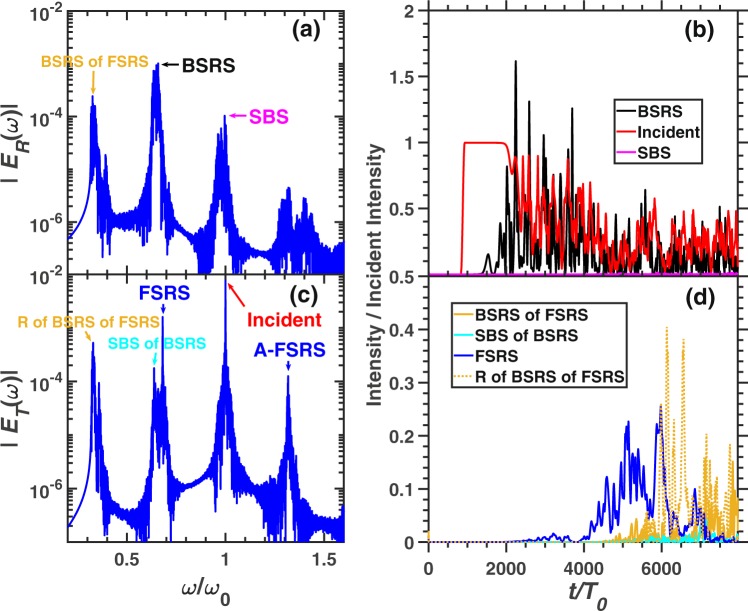
The spectra and scattering rate of each scattering in the condition of case 4. The frequency spectra of (**a**) reflective light electric field ${E}_{R}$ and (**c**) transmitting light electric field ${E}_{T}$. (**b**) The intensity of BSRS $([0.45,0.75]{\omega }_{0})$ and SBS $([0.9,1.1]{\omega }_{0})$ at left boundary, and transmitting light $([0.9,1.1]{\omega }_{0})$ at right boundary. (**d**) The intensity of FSRS $([0.665,0.8]{\omega }_{0})$, SBS of BSRS $([0.45,0.665]{\omega }_{0})$, BSRS of FSRS $([0.2,0.45]{\omega }_{0})$ and reflection of BSRS of FSRS $([0.2,0.45]{\omega }_{0})$. The condition is case 4: ${n}_{e}=0.1{n}_{c},{I}_{0}=3\times 1{0}^{15}W/c{m}^{2},{L}_{x}=5000c/{\omega }_{0}$.

When the electron density is lower than $\sim 0.108{n}_{c}$ in the condition of ${T}_{e}=2.5keV$, the BSRS of FSRS will occur. Figure. 12 demonstrates the spectra and scattering rates of each scattering light in the condition of ${n}_{e}=0.1{n}_{c}$, other conditions are the same as case 3 shown in Fig. 5. Besides SBS, BSRS, SBS of BSRS, FSRS and A-FSRS, the BSRS of FSRS will occur. The electron density corresponding to the critical density of BSRS of FSRS ${n}_{c1}$ is ${n}_{e}=0.1{n}_{c}=0.1/0.32{6}^{2}{n}_{c1}=0.94{n}_{c1}$, which is close to the critical density of BSRS of FSRS. Thus, the reflectivity of BSRS of FSRS will be very strong. We can see that reflectivity of BSRS of FSRS (labelled as “R of BSRS of FSRS”) is stronger than BSRS of FSRS. The average scattering rates are as follows: BSRS: $16.49 \%$, BSRS of FSRS: $0.68 \%$, R of SBS of FSRS: $2.38 \%$, SBS: $0.093 \%$, SBS of BSRS: $0.19 \%$, FSRS: $3.2 \%$, A-FSRS: $0.094 \%$, transmitivity: $38.63 \%$, and absorption: $38.25 \%$. Besides BSRS, the dominant scatterings are R of BSRS of FSRS, FSRS. The SBS and SBS of BSRS are very weak, which can be negligible. Since BSRS of FSRS will develop after FSRS, the FSRS will dominate before $t\simeq 6000{T}_{0}$, and the reflectivity of BSRS of FSRS will dominate after $t\simeq 6000{T}_{0}$. Since BSRS is in the regime of convective instability in condition of ${n}_{e}=0.1{n}_{c},{T}_{e}=2.5keV$, and in the regime of absolute instability in condition of ${n}_{e}=0.2{n}_{c},{T}_{e}=2.5keV$, the BSRS in condition of ${n}_{e}=0.1{n}_{c}$ as shown in case 4 will be obviously weaker than that in condition of ${n}_{e}=0.2{n}_{c}$ (case 3). Thus, the transmitivity in case 4 (Fig. 12) is much larger than that in case 3 (Fig. 5). Large-amplitude BSRS will deplete the pump light in case 3, thus the FSRS excited by the pump light in case 3 is weaker than that in case 4. Since the BSRS with scattering rate $16.49 \%$ in the condition of ${n}_{e}=0.1{n}_{c}$ shown in Fig. 12 is not strong, thus the SBS of BSRS will be very weak. Therefore, in the low electron density region, the SBS of BSRS has little effect on BSRS. When ${n}_{e} < 0.1{n}_{c}$, such as ${n}_{e}=0.09{n}_{c}$, the main rescattering mechanism of BSRS is BSRS of BSRS^34^.

**Figure 13:**
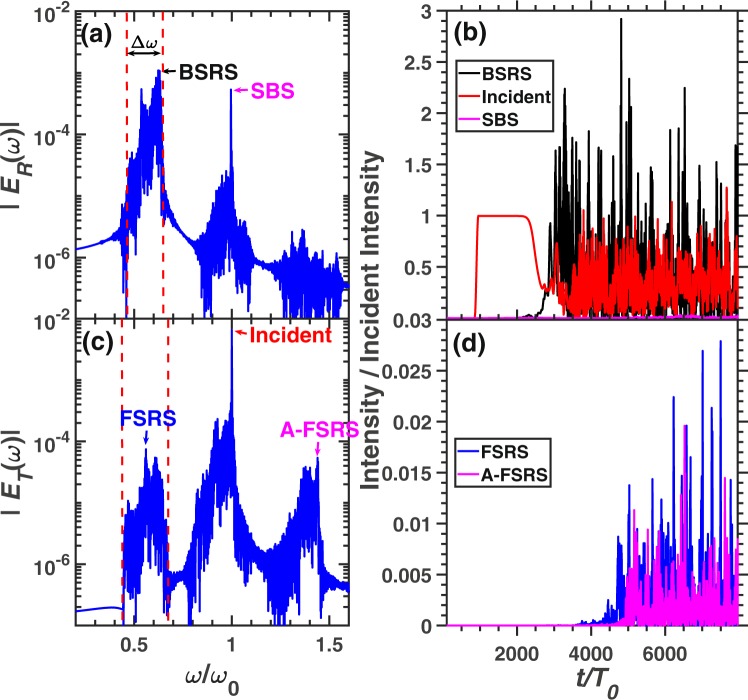
The spectra and scattering rate of each scattering in the condition of case 5. The frequency spectra of (**a**) reflective light electric field ${E}_{R}$ and (**c**) transmitting light electric field ${E}_{T}$. (**b**) The intensity of BSRS $([0.4,0.75]{\omega }_{0})$ and SBS $([0.8,1.1]{\omega }_{0})$ at left boundary, and transmitting light $([0.8,1.1]{\omega }_{0})$ at right boundary. (**d**) The intensity of FSRS $([0.4,0.7]{\omega }_{0})$ and A-FSRS $([1.2,1.5]{\omega }_{0})$. The condition is case 5: ${L}_{x}=5000c/{\omega }_{0}$ with linear density from ${n}_{e}=0.1{n}_{c}$ to $0.2{n}_{c}$, and ${I}_{0}=3\times 1{0}^{15}W/c{m}^{2}$.

 Figure 13 demonstrates the simulation of an inhomogeneous plasma with linear density $0.1{n}_{c}-0.2{n}_{c}$ among plasma scale. Since there exists density gradient, an effective damping from density gradient will exist, the FSRS will be suppressed compared to case 4 and case 3. The average scattering rates are as follows: BSRS: $29.73 \%$, SBS: $0.38 \%$, FSRS: $0.12 \%$, A-FSRS: $0.078 \%$, transmitivity: $41.74 \%$, and absorption rate: $27.95 \%$. The density gradient will decrease the absorption of pump light and enhance the transmitivity. We can see that the transmitivity in case 5 will be larger than those in case 4 and case 3. The inhomogeneous density plasmas will produce wide spectra of BSRS and FSRS as shown in Fig. 13(a,c). The spectrum of SBS of BSRS is also wide, which will overlap with FSRS. Thus, the spectrum of SBS of BSRS can not be distinguished from the FSRS. The BSRS spectrum with bandwidth $\Delta \omega$ can also suppress corresponding rescattering^46^, such as SBS of BSRS. In the same way, the wide-band FSRS spectrum can suppress corresponding rescattering, such as BSRS of FSRS. Thus, the SBS of BSRS and BSRS of FSRS can nearly not occur.

We have also conducted simulation in the condition of fixed ions, which is not shown in this paper. The other conditions are the same as case 2 except the ions are fixed, which is case 6 as shown in Table 1. There only exist BSRS, FSRS, A-FSRS and corresponding reflective lights in the spectra. And the SBS, SBS of BSRS and LDI will not occur. The average scattering rates of scatterings are: BSRS: $46.1 \%$, FSRS: $0.025 \%$, A-FSRS: $0.012 \%$ and transmitivity: $10.16 \%$. Compared to case 2, case 6 illustrates that LDI can suppress BSRS obviously, and also SBS of BSRS may be an important saturation mechanism of BSRS, since there exists no LDI and SBS of BSRS in the case 6.

## Conclusions

In conclusions, a rescattering of BSRS by SBS has been researched by both 1D Vlasov simulations and 2D PIC simulations. The novel rescattering mechanism SBS of BSRS is found both in short-scale and long-scale 1D and 2D systems. The SBS of BSRS will be stronger than SBS, which may be an important saturation mechanism of BSRS in regime of absolute instability for BSRS. Especially, in small scale plasmas, the SBS of BSRS will be stronger than FSRS. Besides LDI and laser energy absorption, SBS of BSRS will reduce BSRS, thus saturating BSRS. And density gradient can on one hand reduce FSRS and absorption of pump light, thus increasing transmitivity, and on the other hand reduce SBS of BSRS and BSRS of FSRS by wide-band frequency. These results are important to increase transmitivity of pump light and reduce BSRS in ICF, and the novel rescattering mechanism produces a light with new frequency, which may be taken used of in optics.

## Methods

A one dimension (1D) relativistic Vlasov-Maxwell code^47,48^ is taken used of to verify the SBS of BSRS. Since H plasmas is common in ICF hohlraum, it is taken as a typical example in our simulation. The electron temperature is ${T}_{e}=2.5keV$ and the ion temperature is ${T}_{i}=1/3{T}_{e}$. The incident laser is linearly polarized laser with wavelength ${\lambda }_{0}=0.351\mu m$. The incident laser is a plane light with a single mode and a very narrow spectral line-width. The spectral line-width of incident laser can be negligible. The spatial scale is [0, ${L}_{x}$] discretized with spatial step $dx=0.1c/{\omega }_{0}$. And the spatial length is ${L}_{x}=500c/{\omega }_{0}$ or ${L}_{x}=5000c/{\omega }_{0}$ with $2\times 5 \% {L}_{x}$ vacuum layers and $2\times 5 \% {L}_{x}$ collision layers in the two sides of plasma boundaries. The strong collision damping layers are added into the two sides of the plasma boundaries to damp the electrostatic waves such as LWs and IAWs at the boundaries. The velocity scale is discretized with ${N}_{v}=512$ grid points. The total simulation time is ${t}_{end}=5\times 1{0}^{4}{\omega }_{0}^{-1}\simeq 7.96\times 1{0}^{3}{T}_{0}$ discretized with ${N}_{t}=5\times 1{0}^{5}$ and time step $dt=0.1{\omega }_{0}^{-1}$. Other simulation parameters such as electron density ${n}_{e}$, pump laser intensity ${I}_{0}$ and spatial scale ${L}_{x}$ are listed in Table 1.

A two dimension (2D) particle-in-cell (PIC) code EPOCH^49,50^ is used to research SRS and SBS of BSRS. The electron temperature is ${T}_{e}=2.5keV$ and electron density is ${n}_{e}=0.2{n}_{c}$, where ${n}_{c}$ is the critical density for the $3\omega$ pump light. The ion temperature is ${T}_{i}=1/3{T}_{e}$. The linearly polarized pump laser with wavelength ${\lambda }_{0}=0.351\mu m$ is taken. The spatial scale is [0, ${L}_{x}$]$$\ \times \ $$[$-{l}_{y}/2$, ${l}_{y}/2$] discretized with spatial step $dx=0.1{\lambda }_{0}$ and $dy=0.1{\lambda }_{0}$. And the $x$-direction spatial length is ${L}_{x}$ with $2\times 10{\lambda }_{0}$ vacuum layers in the two sides of plasma boundaries, and $y$-direction spatial length is ${L}_{y}=2{l}_{y}=40{\lambda }_{0}$. The plasma locates at the center with simulation length ${L}_{sim}={L}_{x}-20{\lambda }_{0}$ and $200$ electrons or ions per cell in the short scale 2D simulations with ${L}_{x}=100{\lambda }_{0}$ or $200{\lambda }_{0}$. Open boundary condition of laser and thermal boundary condition of particle are used in the $x$-direction and periodic boundary is used for both laser and particle in $y$-direction. The total simulation time of short scale 2D PIC simulations is ${t}_{end}=3\times 1{0}^{3}{T}_{0}=3.51ps$, where ${T}_{0}=1.17fs$ is the period of pump light. The output snapshot time of simulation is $d{t}_{snapshot}=0.1{T}_{0}$. Due to limitation of calculation, a long time and large scale system laser plasma interaction is researched by 2D PIC simulation with less particle per cell and 1D PIC simulation with high precision. The other parameters are the same as the simulations from short scale 2D PIC simulation. Except that ${L}_{x}=1000{\lambda }_{0}$ with $2\times 10{\lambda }_{0}$ vacuum layers in the two sides of plasmas boundaries and ${t}_{end}=1.5\times 1{0}^{4}{T}_{0}=17.55ps$ are taken in large scale 2D PIC simulation, and 2 electrons or ions distribute in a cell. While in large scale 1D simulations, ${L}_{x}=2020\mu m$ with $2\times 10\mu m$ vacuum layers in the two sides of plasma boundaries and ${t}_{end}=1.5\times 1{0}^{4}{T}_{0}=17.55ps$ are taken.
